# Co-tailoring a depression intervention with and for Black women survivors of intimate partner violence

**DOI:** 10.21203/rs.3.rs-11040477/v1

**Published:** 2026-10-12

**Authors:** Bernadine Y. Waller, Dawn Goddard-Eckrich, Chiamaka Chidi, Ana Stefancic, Michelle Richardson-Ridley, Whittni L. Holland, Mosinmiloluwa Adeyemo, Temiloluwa Adeyemo, Danielle Williams, Sidney H. Hankerson, Rachel C. Shelton

## Abstract

Background Black women experience intimate partner violence (IPV)-related mortality and morbidity at disproportionate rates. With an adverse circuitous relationship between IPV-related femicide and poor psychological health, there is renewed urgency to improve survivors’ mental wellbeing. Still, a dearth of depression interventions have been tailored to meet Black survivors’ culturally specific needs. Purpose To understand the psychosocial needs of Black women IPV survivors and begin tailoring a depression intervention with and for them. Methods Forty-six in-depth, semi-structured interviews were conducted across two studies. The first study included data collection among 30 Black women who were help-seeking for IPV-specific services. Theoretical sampling was utilized. Data collection occurred until saturation. Constructivist grounded theory methodology was employed. The second study included data collected from one intervention workgroup. The day-long workgroup drew from a sample of Black survivors, clergy, and providers (n = 16). The PRISSMA framework guided the workgroup process, and the first two steps of the ADAPT-ITT Model were completed with the goal of selecting and begin tailoring an intervention for depression. Results Nine phases identifying Black survivors’ psychosocial help-seeking were found in the data. The first phase identified depression as a pressing concern. This informed the necessity of tailoring an intervention for depression. The workgroup opted to tailor interpersonal counseling for depression, infusing faith and spirituality with psychoeducation within the adapted intervention. Conclusions Centering Black women’s needs is critical to the continuum of work necessary to improving the mental health and overall wellbeing of Black survivors. Sarah Waller’s Help-Seeking model is the first to identify Black IPV survivors’ needs. The second study is the first to begin tailoring an intervention for depression with and for Black women IPV survivors.

